# Whole Body Microwave Irradiation for Improved Dacarbazine Therapeutical Action in Cutaneous Melanoma Mouse Model

**DOI:** 10.1155/2013/414816

**Published:** 2013-11-26

**Authors:** Monica Neagu, Carolina Constantin, Diana Martin, Lucian Albulescu, Nicusor Iacob, Daniel Ighigeanu

**Affiliations:** ^1^Immunology Department, Immunobiology Laboratory, “Victor Babes” National Institute of Pathology, 99-101 Splaiul Independentei, sector 5, Bucharest 050096, Romania; ^2^National Institute for Laser, Plasma and Radiation Physics, 409 Atomistilor Street, Magurele 077125, Romania; ^3^Department of Infectious Diseases and Immunology, Virology Division, Faculty of Veterinary Medicine, Utrecht University, Yalelaan 1, 3584 CL Utrecht, The Netherlands

## Abstract

A cutaneous melanoma mouse model was used to test the efficacy of a new therapeutical approach that uses low doses of cytostatics in conjunction with mild whole body microwave exposure of 2.45 GHz in order to enhance cytostatics antitumoral effect. *Materials and Methods.* A microwave exposure system for C57BL/6 mouse whole body microwave irradiation was designed; groups of 40 mice (males and females) bearing experimental tumours were subjected to a combined therapy comprising low doses of dacarbazine in combination with mild whole body irradiation. Clinical parameters and serum cytokine testing using xMAP technology were performed. *Results.* The group that was subjected to combined therapy, microwave and cytostatic, had the best clinical evolution in terms of overall survival, tumour volume, and metastatic potential. At day 14 the untreated group had 100% mortality, while in the combined therapy group 40% of mice were surviving. Quantifying serum IL-1**β**, IL-6, IL-10, IL-12 (p70), IFN-**γ**, GM-CSF, TNF-**α**, MIP-1**α**, MCP-1, and KC during tumorigenesis and therapy found that the combined experimental therapy decreases all the inflammatory cytokines, except chemokine MCP-1 that was found increased, suggesting an increase of the anti-tumoral immune response triggered by the combined therapy. The overall metastatic process is decreased in the combined therapy group.

## 1. Introduction

Malignant melanoma (MM) is one of the most aggressive human cancers, since a few mm thick tumors have full potential to kill the host in more than 80% of the cases [1]. Besides the surgical elimination of the primary tumor, there is no other effective cure for MM [1, 2]. MM is resistant to ionizing radiations (radiotherapies) as well as to conventional chemotherapies. The combination of ionizing radiation as well as nonionizing radiation (such as microwaves) with other therapies is reported as a promising strategy in cancer therapy [2]. 

 Microwaves (MW) are presently used or under study for therapeutic applications in areas such as cardiology, urology, general surgery, ophthalmology, and oncology. MW is used as well for organ imaging in the clinical diagnostic of cancer [3].

In the last years there is a revival of therapeutical possibilities to use MW in oncology, in both animal *in vivo* models studies and in clinical trials. Low-intensity microwave radiation used in animal model inoculated with sarcoma 45 cell line has shown that in 50% of animals' tumor growth and partial regression was obtained. The treatment was efficient due to the actual destruction of tumors and accumulation of antitumoral immune cells [4].

 Recent technical study revealed that MW can generate *in vivo* a larger ablation zone compared with multipolar radiofrequency (RA) [5]. When used in actual patients presenting hepatocellular carcinoma as liver metastases, MW has the potential to decrease local recurrence when compared to RF-based therapy [6].

 A study comprising results gathered for 10 years regarding microwave therapy in scapular tumors has shown that *in situ* microwave therapy for malignant tumors in the scapula can lead to reliable clinical effects and patient acceptability [7]. Treatment of bile duct carcinoma with thin coaxial antenna was recently showing the relation between tissue coagulation size and radiation power shown [8].

 Thoroughly reviewed in 2010 [9] the hyperthermia-based therapy, used individually or as additional therapy, can adjoin the surgery for inoperable tumors, can treat relapsed patients without increasing toxicity, and so on. In this seminal review results of phase III randomized trials were shown. The conclusion of this study is that a microwave generator can induce a superficial hyperthermia or a radiofrequency applicator can enter more deeply into the tissues. MW appears to be the fourth treatment pillar beside surgery, radiotherapy, and chemotherapy [10].

 MW, as nonionizing radiation, interacts with matter by different physical action, interaction, that is related to their physical parameters: frequency, polarization, modulation, power density, field uniformity, and temperature. The interaction is dependent on the properties of biological materials, expressed in terms of the complex relative permittivity *ε* = *ε*′ − *jε*′′ with loss tangent tan*δ* = *ε*′′/*ε*, biological sample parameters (nature, size and geometry of samples, and sample orientation relative to polarization), and environmental factors (temperature, humidity) [11, 12]. Since the quantum energy of the MW is not sufficient to cause atom ionization [13], its biological effects on tissues may be explained by thermal and nonthermal mechanisms [14]. From this complex array of possible effects, only one biological effect of MW is well known, namely, heating. The effect of MW is explained especially by their heating property on the polar or polarizable molecules of biological systems considered as water dominated dielectrics richly endowed with electrolytes and intricately packaged polar and nonpolar molecules [3, 11, 12, 15]. Some MW biological effects occur over a limited range (“windows”) of frequencies or modulations and other biological effects have been reported to occur in multiple dose or intensity ranges, referred to as intensity windows, instead of showing classical ionizing radiation dose-response relationships [11, 14, 16–20]. These effects include, altered cell proliferation, cell membrane receptor-mediated events, alterations of the membrane channels, and many more cellular events. 

 In cancer treatment, one of the major side effects of chemotherapy is that it can suppress natural killer (NK) cell activity and enhance tumor evolution and metastasis. It was shown that the millimeter-waves (MMWs) irradiation (42.2 GHz) can inhibit tumor metastasis enhanced by cyclophosphamide (CPA), a known anticancer drug [21]. CPA was reported to induce a fivefold enhancement of the tumor process, which was significantly reduced when CPA-treated animals were irradiated with MMWs. MMWs also increased NK cell activity suppressed by CPA, suggesting that the observed reduction of tumor metastasis is mediated through activation of NK cells [21]. 

 Whole body hyperthermia, a procedure in which the body temperature is elevated by MW exposure to 41–43°C, has been investigated as a treatment for cancer, most commonly as an adjunct to radiotherapy (thermoradiotherapy) or chemotherapy (thermochemotherapy) [22–24]. In 2011, it was reported that when combining microwave-based whole body hyperthermia with cytostatics cis-diaminedichloroplatinum (CDDP) and dacarbazine (DTIC) a good antitumoral response was obtained, namely, inhibition of melanoma tumor cell proliferation, reduced neovascularization, and an increase in the specific immune responses [25].

 The transient permeable state of the cell membrane obtained by applying short, intense electric pulses, designated as “cell electroporation,” was intensively studied both *in vitro* and *vivo, *technique that aimed to introduce nonpermeable molecules into living cells. European clinical experience gathered in the last years regarding this therapy showed good therapeutical results in skin and soft tissue tumours [26–30]. The reported data analysis have showed that proper whole body irradiation with MW enhances the tumoricidal effects of chemotherapy, overcomes acquired drug resistance, and stimulates certain components of the immune system involved in the antitumoral action. Our *in vitro* experiments, on melanoma cell lines [31], have suggested that similarly to the cell membrane electroporation effect, the MW exposure could be able to increase the drug delivery into melanoma cells only at high enough Specific Absorption Rate (SAR) values, that is, at high electric field strength values of the MW electric field component and appropriate Specific Absorption (SA) that overcome the temperature rise over 37-38°C. 

 DTIC, the only FDA-approved cytostatic for metastatic melanoma [32] is an imidazole carboxamide derivative with several proposed mechanisms of action [33]. Besides the secondary effects, there are several down-falls in DTIC treatment, one being the fact that high dose of DTIC can select a more aggressive form of melanoma phenotype [34]. Overall the main draw back in cutaneous melanoma therapy is its high resistance to cytostatics. Taking into account the DTIC toxicity the main goal of this work was to investigate the effects of small doses chemotherapy in conjunction with total body MW irradiation. Thus, we aimed to enhance tumour sensitivity to cytostatic and enlarged the panel of efficacious therapies using a mouse experimental model. We used low doses of cytostatics combined with MW irradiation in order to enhance drug sensitivity of skin tumours. In terms of DTIC concentration prior published studies in mice models have shown DITC doses as high as 80 mg/kg, with a 5-day administration [35] or 60 mg/kg administration [36]. Thus, we have used a low dose of DTIC, namely, 5 mg/kg/mice. The survival rate of mice, tumour volume, and soluble cytokine monitorization were followed during therapy. Using concomitant detection through multiplexing techniques we have tested cytokines/chemokines highly involved in immune processes triggered by tumour development. The serum pattern of cytokine production was used as efficacy markers for the skin melanoma experimental therapy. 

 In the last 15 years very few papers were published regarding cutaneous melanoma animal models for experimental therapy with MW. In our model using this combined therapy we decreased the concentration of therapeutical doses of DTIC increasing its clinical efficacy.

## 2. Material and Methods

### 2.1. Murine Experimental Model

We have used an established animal model for developing cutaneous melanoma [37], namely, the *in vivo* model of subcutaneous growth of B16 melanoma. Female and male C57BL/6J mice purchased from Jackson Laboratory (Bar Harbor, ME) were maintained in standard conditions in “Victor Babes” National Institute of Pathology Animal Husbandry. Recognized principles of laboratory animal care were followed [38] in the framework of *EU Directive 2010/63/EU for animal experiments*. All animal protocols were approved by “Victor Babes” National Institute of Pathology Animal Care and Use Committee. 

 All mice 6 weeks old having a mean weight of 20  ±  2 g entered the experimental procedures in groups consisting of 20 males and 20 females. The groups that were supposed to develop the skin melanoma were subcutaneously inoculated with 1  ×  10^5^ B16F10 (ECACC 92101204) melanoma cell lines/mouse. In the 7th day after inoculation mice were treated intramuscularly with low doses of DTIC (5 mg/kg/mouse) for 5 days at intervals of 24 h between treatments and subjected to whole body irradiation with MW (SAR = 1.63 W/mouse/day and SA = 74.98 J/mouse/day) in the below described original apparatus. Mice were retroorbitally bled at day 0, day 7 from B16 inoculation, and after treatment. Blood was subjected to serum harvesting and afterwards stored at −70°C until testing.

 The following groups were tested.

- Control mice, tumor free animals, divided in the following groups: untreated (control), subjected to MW irradiation, subjected to combined therapy (MW and DTIC), and subjected only to DTIC.
- Mice bearing B16F10 melanoma tumor untreated.
- Mice bearing B16F10 melanoma tumor divided in the following groups: subjected to MW irradiation, subjected to combined therapy MW, and DTIC and subjected only to DTIC.

MW irradiation was applied concurrently with, prior to, or following DTIC administration. The presented results displayed the best clinical outcome and irradiation followed DTIC inoculation.

### 2.2. Experimental Procedure: Total Body Irradiation

**Figure 1:**
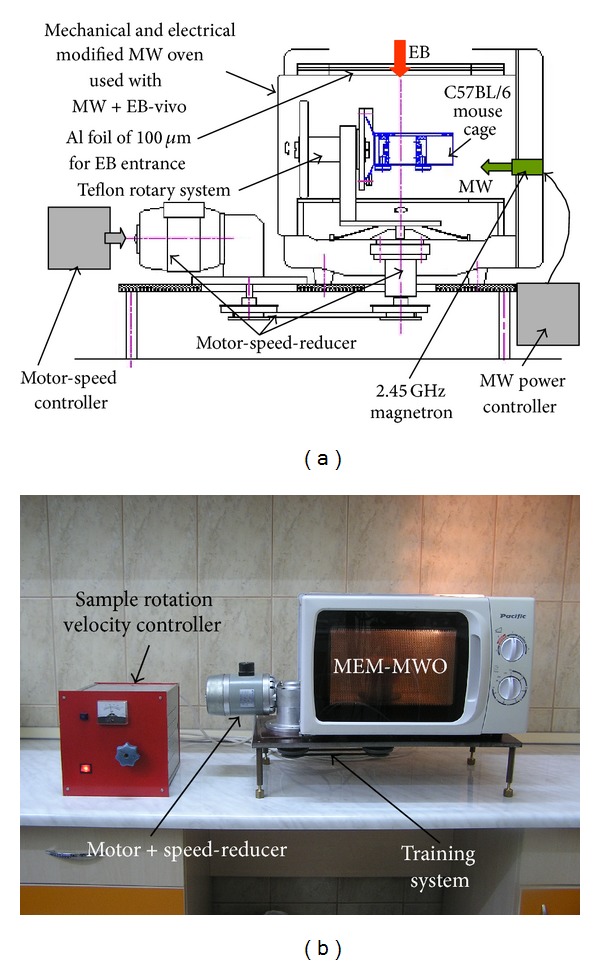
(a) Schematic drawing of the MEM-MWO; (b) photograph of the MEM-MWO.

**Figure 2:**
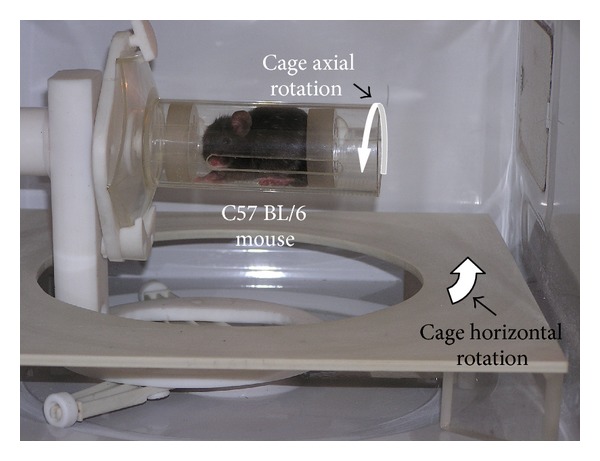
Photograph of MEM-MWO internal configuration used with MWES.

The MW exposure system (MWES) used for C57BL/6 mouse in a whole body irradiation procedure with microwaves of 2.45 GHz is an innovative and flexible experimental installation (special designed for separate, successive and combined irradiation with MW, and accelerated electron beam) that was previously described and reported in [31]. For better understanding of experiments performed in the frame of this work, we decided to render several features of MWES. It consists mainly of a radiation exposure chamber (REC) and a microwave source of 2.45 GHz with adjustable output power (0–50 W), generated as 10 ms pulses at 50 Hz repetition rate, as REC the multimode rectangular cavity of a proper mechanical and electrical modified MW oven (MEM-MWO) is used. In this installations the conventional operation of 2.45 GHz oven magnetron supplied by an L.C. single-phase-half-wave doublers (L.C. HWD) was modified in order to permit the use of a manually or PC-controlled electronic regulator for the MW power adjustment and remote control [39]. The magnetron main power units consisting of a high voltage diode, a high voltage capacitor, and a high voltage anode transformer (HVAT) are similar to the units used for the conventional magnetron supplying system. Modification consisted in the use of a separate transformer for the filament supply and of a triad controlled regulator added to the HVAT primary circuit. Also, several electronic units are added for MW exposure time presetting as well as for magnetron peak and average current measurement. Another feature added to the MWES operation was obtained by modifying the geometry and rotation velocity of the sample rotary system, as shown in Figures 1(a), 1(b), and 2. 

By this procedure the sample rotation velocity can be modified from one rotation per second to one rotation per 30 seconds depending on the desired dose at certain MW power levels. For the experiments with MWES, the C57BL/6 mouse is placed into a special designed cylindrical cage.  Figure 2 shows the photograph of this cage containing inside a C57BL/6 mouse. The C57BL/6 mouse cage is made up from a marked cylinder of 250 mL, PMP 2574 type cut at 112 mm from its sole. Two Teflon pistons with aeration apertures assure the mouse immobilization during radiation exposure. During the radiation exposure time the mouse cage can perform two rotation motion types: in the horizontal plane and around its axis (Figure 2). 

 During one horizontal rotation the mouse cage accomplishes two axial rotations. Horizontal motion transmission to the mouse cage is performed by a Teflon arm fitted to the upper end with an aperture in which a Teflon axle is rotating. On the one Teflon axle end a mouse cage is mounted and on the other end a Teflon friction wheel that is in permanent contact with a fixed platform that generates the cage axial rotation. The desired radiation exposure homogeneity and reproducibility of the C57BL/6 mouse in the cage is obtained by presetting the exposure time so that each mouse is to perform only complete rotations (one, two, or more) inside MEM-MWO multimode cavity during irradiation process. The mouse cage motion starts and interrupts simultaneously with MW switch on and switch off, respectively. 

**Figure 3:**
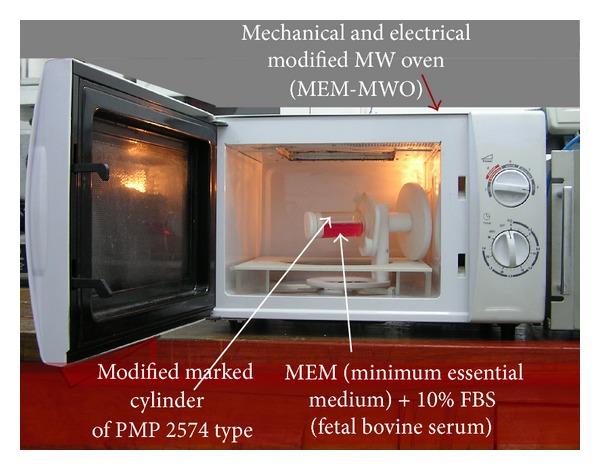
Photograph of the MEM-MWO used for an approximate evaluation of the MW power amount absorbed by a mouse.

 For an approximate evaluation of the MW power amount absorbed by a mouse of certain mass, we determined the dependence of the absorbed MW power by different distilled water and culture medium samples placed in the same geometrical configuration as the mouse's cage. The experimental arrangement (EA) is shown in Figure 3. Because we selected for experiments mice with an initial mass of 20  ±  2 g, it was assumed that during treatment the mouse mass may increase up to 30 g; the sample volumes used in the experiments performed with the installation shown in Figure 3 were 20, 25, and 30 mL. 

 The proper correlation between magnetron average current and MW power as well as between MW power and SAR characterizing MW exposure is a very difficult procedure because the SAR depends strongly on geometry and electromagnetic properties of exposed material as well as on environmental factors that are variable and cannot be well controlled during treatment especially in *in vivo* conditions. Also, only a small amount of offered MW energy is absorbed by small sample volumes [40]. Different sample volumes absorb different MW energies from the same offered MW energy in the exposure applicator. In our opinion SAR and SA could be given by W per mass of sample mass and J per mass of sample, respectively, pointing out each time the sample nature, geometry, applicator type, and exposure geometry.

**Figure 4:**
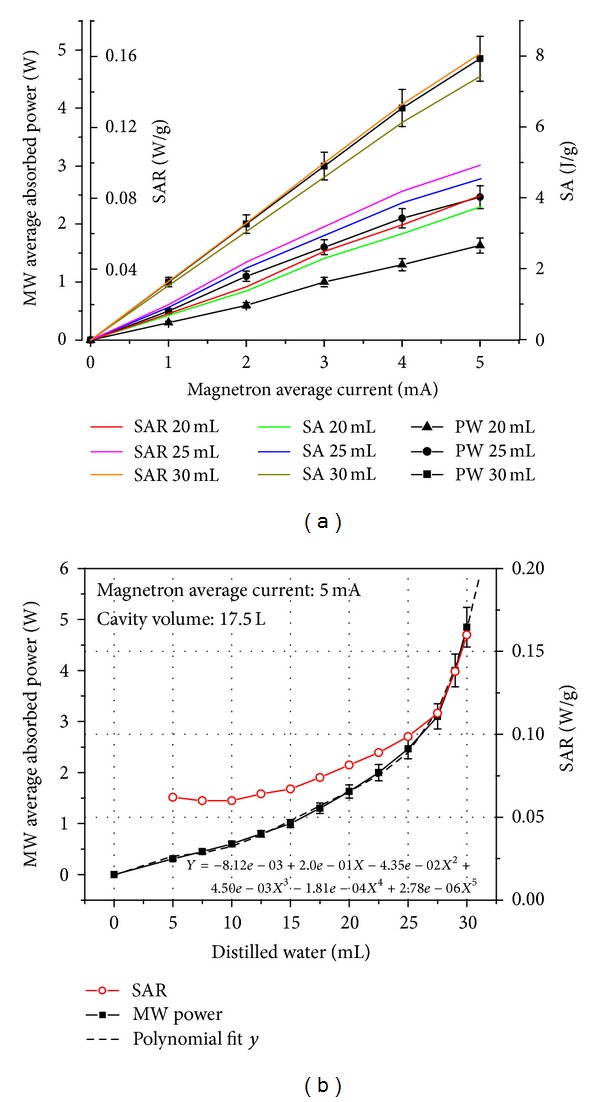
(a) *P*_*A*_, SAR and SA versus magnetron average current for distilled water samples; (b) *P*_*A*_ versus distilled water volume.

 The dependence of MW absorbed power (*P*_*A*_), SAR, and SA versus magnetron average current for distilled water samples is presented in Figure 4(a). 

As seen in Figure 4, the MW absorbed power, SAR, and SA depend strongly on water volume of samples (20, 25, and 30 mL) at the same value of magnetron average current. The usage of homogenous animal mass at the procedure start was essential due to SAR and SA parameters that can fluctuate in regard to each mouse mass. During treatment over many days, this need is very difficult to be kept as well as in the case of mice bearing cutaneous melanoma. In these circumstances, the SAR and SA will increase or decrease depending on the evolution of each mouse mass and tumor volume. As a consequence, the SAR and SA cannot be well controlled during microwave irradiation. The only parameters that can be controlled during microwave irradiation are magnetron average current and MW exposure duration that is correlated with the number of complete rotations of the mouse cage in the horizontal plane and around its axis. Finally we set out to use in our experiments a magnetron average current of 5 mA and a MW irradiation time of 46 s (i.e., two complete horizontal and four complete axial rotations of the cage with C57BL/6J mouse into radiation exposure chamber). The corresponding values for SAR and SA for mice of 20  ±  2 g initial mass are SAR = 1.63 W/mouse (0.0815 W/g) and SA = 74.98 J/mouse (3.749 J/g). These values were established to satisfy our demands that the estimated SAR is as high as possible, while the variation of mouse skin surface temperature during MW exposure is kept in the range of 2–5°C. For these experimental conditions the obtained results were presented in Figure 4(b) and Table 1. As seen in Figure 4(b) the SAR variation versus sample volume has a lower growth rate than *P*_*A*_ experimental determined versus water volume increasing up to 30 mL. This demonstrates that SAR increasing due to the increasing *P*_*A*_ is partially compensated because its variation is inversely proportional to sample volume. Also, this suggests that although the MW absorbed power could increase or decrease during MW exposure due to the mouse mass variation, assessed SAR expressed as *P*_*A*_ per mouse mass (W/g) will have a lower variation rate than *P*_*A*_ as is shown in Table 1.

As seen in Table 1, the AM (measured average mass of all mice, female and male, over all treatment days with MW) is 23.2034  ±  0.1630 g, corresponding *P*_*A*_ (determined from polynomial fit “*y*” plotted in Figure 4(b)) is 2.0471  ±  0.02257 W and estimated SAR is 0.0882  ±  0.0005 W/g. This example demonstrates that although AM and *P*_*A*_ are different values compared with initial values (20 g and 1.63 W) from first treatment day with MW, the SAR value of 0.0882 W/g, averaged over all treatment days, is close to its initial value of 0.0815 W/g.

**Figure 5:**
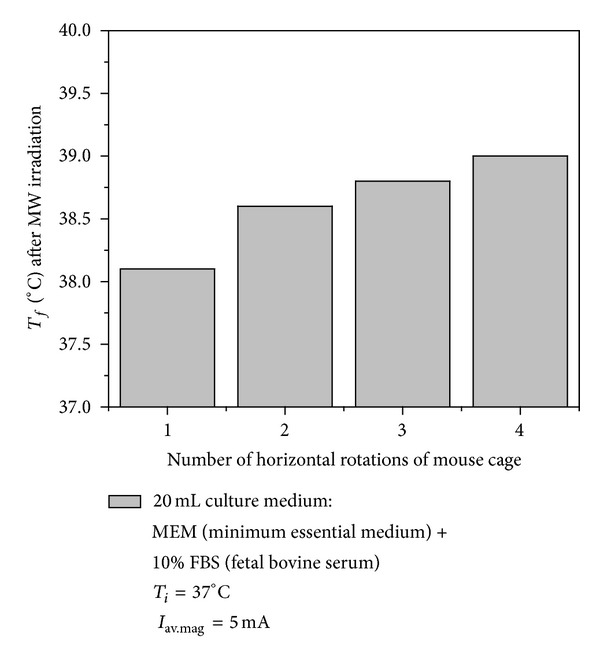
Final temperature (*T*_*f*_) versus number of complete horizontal rotation of mouse cage during MW irradiation with 5 mA magnetron average current (*I*_av.mag_).

Figure 5 shows final temperature (*T*_*f*_) versus number of complete horizontal rotation of mouse cage during MW irradiation with 5 mA magnetron average current for culture medium sample of 20 mL. 

 The mouse skin surface temperature was measured before and after MW exposure with a non-contact-type infrared thermometer. The mouse surface temperature increased in the range of 3–5°C for healthy mice group and 1.5–3°C for tumor-bearing mice group. The MW irradiation in conjunction with DTIC administration in melanoma bearing mice increased by about 2°C.

### 2.3. Clinical Parameters

Tumor volume was measured and expressed in mm^3^ according to Egorov [41] after the following formula:

(1) $$\begin{array}{c} V=\frac{\pi }{6}\times \left(\text{Length}\right)\times \left(\text{Width}\right)\times \left(\text{Height}\right). \end{array}$$

Results are presented as mean ± SD mm^3^.

*Postmortem* necropsies were performed for confirming the presence and extent of neoplastic growth. The sampled tissues were subjected to histological analyses. Survival of mice was monitored until the animals' quality of life was not drastically affected and they were euthanized when required.

### 2.4. Serum Cytokine Testing Using xMAP Technology

Using Mouse Cytokine/Chemokine Lincoplex Kit (Cat. number MCYTO-70K) and Luminex 200 concomitant serum cytokine were quantified: IL-1*β*, IL-6, IL-10, IL-12 (p70), IFN-*γ*, GM-CSF, TNF-*α*, monocyte chemoattractant protein (MCP-1), macrophage inflammatory protein (MIP-1), and keratinocyte-derived chemokine (KC) after producer's recommendations.

 The calibration curves were obtained using the provided standards and the method accuracy was analyzed using the registered high and low controls provided by the producer and laid within the recommended ranges. Results are presented as indexes of the actual pg/mL serum concentrations compared to each proper control (mean ± SD).

For statistical analysis the unpaired Student's *t*-test was used.

## 3. Results

### 3.1. Clinical Parameters in Combined Therapy: Cytostatics and Whole Body Irradiation

**Figure 6:**
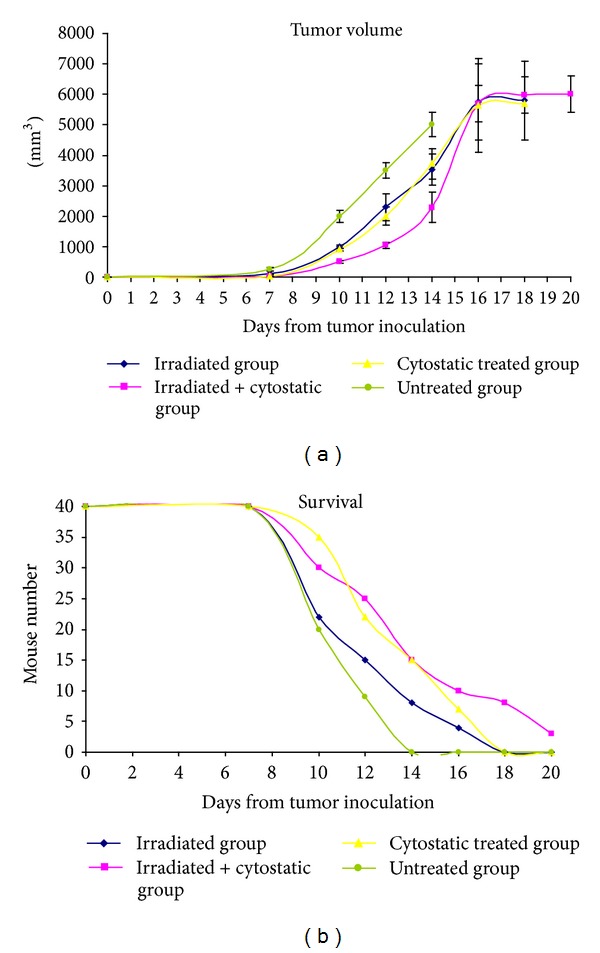
Tumor volume (a) and survival rate (b) in mice experimental model.

Measuring the tumor volume, we have observed that immediately after starting the therapy the groups just “split” in terms of tumor volume (Figure 6(a)). Therefore, as expected, the untreated group has had the highest increase in tumour volume and the increase had the highest rate, while the groups treated with irradiation or only cytostatics have had a similar development. Immediately from the beginning of the experiment, the group subjected to both therapeutical approaches has had a lower volume and a decreased rate of tumour development. At day 14, all mice from the untreated groups have died (Figure 6(b)) and the *postmortem* necropsies and pathology tests have shown, as expected, massive melanoma metastases in all organs, with an increased burden on the brain, lung, pleural membrane, liver, kidney, adrenal glands, lymph nodes, and muscles. The tumor volume of animals treated with cytostatic or irradiated increased at the same rate and, at day 18, all the animals from these groups have died. The tumour of animals subjected to both treatments also had an increased volume, but they were still surviving at day 20 and were euthanized. No significant differences were registered in terms of tumour volume or survival rates between males and females.

Survival rate matches in a good manner the tumour volume increase especially in the untreated group. At day 14 when the untreated group had 100% mortality, in the combined therapy, 40% of mice were surviving. At day 18, whether treated only with the cytostatic or only irradiated, all mice were dead while 25% of the combined therapy group was surviving. This group although bearing tumours as large as 1/3 of their body volume had low extent metastasis. Overall metastasis in combined treated therapy group had lower organ extent compared to controls and/or singular therapy groups.

### 3.2. Serum Cytokines in Mice Subjected to Combined Therapy

In order to find serum cytokines that can be in the future used as disease or therapy monitoring indicators we have quantified them during clinical evolution. The concentration ranges of serum cytokines varies from 0 to 10,000 pg/mL; thus, we have presented the indexes calculated to each subsequent control. 

**Figure 7:**
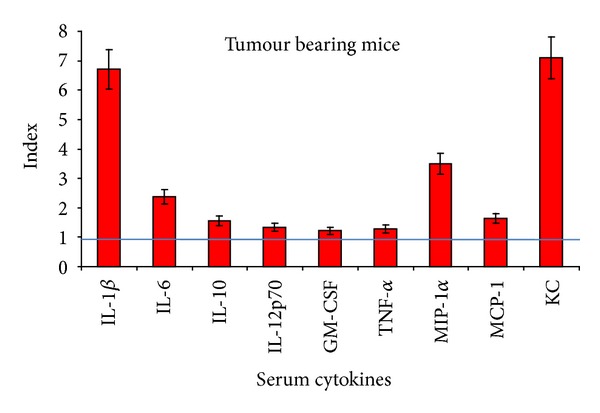
Serum cytokines in tumour bearing animals—index calculated in comparison to control group (mean ± SD); straight line depicts control value.

 After 7 days of B16F10 melanoma cell line postinoculation, animals displayed higher serum concentrations of the tested proinflammatory cytokines. This result was to be expected (Figure 7), and overall we have a 7-fold increase for serum IL-1*β* and for KC, while MIP-1*α* and IL-6 displayed a 4-fold and 2-fold increase, respectively. It seems that in tumor bearing animals, soluble KC is a parameter that is steadily increasing in the serum. One probable explanation is that melanoma cells can intensively secrete KC [42]; therefore, an on-going tumoral process can be associated with an increase in serum KC. 

**Figure 8:**
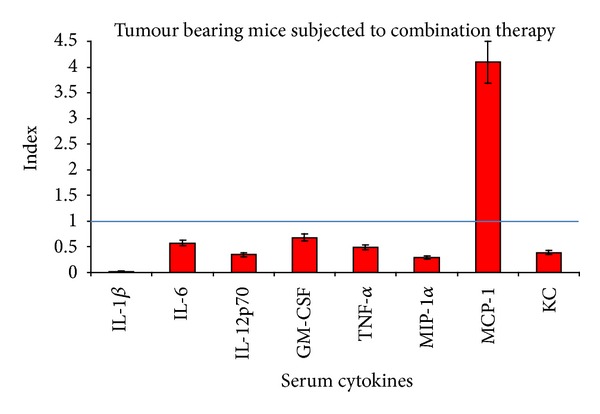
Serum cytokines concentrations in tumour bearing animals subjected to combined therapy—index calculated in comparison to untreated group (mean ± SD); straight line depicting cytokines values in untreated tumour bearing animals.

 In the combined therapy groups (Figure 8), after 11 days of therapy, we obtained a clear decrease of inflammatory cytokines. Only the chemotactic molecule, MCP increases statistically significant, while the other cytokines remained under the ranges detected in nontreated group. MCP is a chemokine involved in attracting and activating both innate and adaptive immune cells and finding it increased is a beneficial sign for the combined therapy antitumor action [43]. 

## 4. Discussion

In a previous study that used MW combined with another cytostatic, cyclophosphamide (CPA) [44], it was shown that MW can restore cytokine production that is suppressed by the drug. The study showed that when used with CPA, MW did not present significant clinical improvement. While CPA acts by adding an alkyl group to the guanine base of DNA, for DTIC several mechanisms of action were proposed. As it has a structure similar to a purine it can inhibit DNA synthesis by acting as a purine analog. Moreover, it can act as CPA, namely, acting as an alkylating agent or it can interact with SH groups. We cannot rule out that all these mechanisms could be involved *in vivo*.

 The cytokines that we have tested have different functions and in tumour development they intervene at different stages. It was reported that MCP-1 favors tumor angiogenesis and early tumor growth by inducing TNF*α*, IL-1*α*, and VEGF by TAMs [45]. Only one recent publication reports the quantification of serum MCP in melanoma bearing mice [46]. In melanoma bearing mice the serum levels of MCP-1, MIP-1*β*, MCP-3, and inducible protein-10 (IP-10) was found significantly increased compared to controls. A report published few years ago demonstrated that melanoma cells cultured in presence of IFN-*γ* or TNF-*α* secreted KC [42]. This finding is more interesting when we add that in tumor bearing animals this chemokine was found significantly increased and after the combined therapy the level of KC decreases considerably.

 Investigating the levels of circulating cytokines, IL-1*β*, 6, 12 p70, GM-CSF, TNF-*α*, MIP-1*α*, and KC decreased statistically significantly after combined therapy, while IL-10 was insensitive. MCP-1 proved to have a different behaviour compared to the other circulating tested molecules out of all the tested one, only MCP-1 increased in relation to combined therapy. The possibility of monitoring by means of cytokine serum level the evolution of melanoma was recently published by us in longitudinal melanoma patients followed up for several years [47], thus opening the possibility to enlarge the panel of biomarkers predictors in melanoma therapy.

 Another noninvasive new therapy, high intensity focussed ultrasound (HIFU), has gained a large amount of interest due the fact that it provides adequate heating to the full tumor volume, in particular for deep seated tumors, and allows noninvasive tissue heating with high spatial accuracy (~mm). Also, the HIFU combination with classical cancer therapies such as chemo- or radiation therapy, as well as immunotherapy, is attracting growing clinical interest [48]. However, in our opinion, “laser-initiated hyperthermia by heat shock using nanoparticles (NPs)” (such gold NPs) will have a higher potential use in nanomedicine for cancer therapy. These nanoparticles can absorb laser light strongly and then rapidly convert this energy into heat, allowing for the selective destruction of cancer cells at laser energies not sufficient to harm surrounding healthy cells. When such NPs are conjugated to cancer antibodies or other cancer targeting molecules, the cancer cells selectively labeled with those nanoparticles can be easily detected under a simple microscope, due to their strongly enhanced light scattering properties [49]. HIFU therapy has been used in Japan [50], but in other countries this therapy has been reported with contradictory results, sometimes failing [51] and sometimes showing improved results in secondary hyperparathyroidism treatment in patients with chronic kidney disease [52]. More radical negative results were published this year when all the prostate cancer patients treated with HIFU relapsed after approximately 6 years after HIFU treatment [53]. The conclusion of the results published until now using HIFU is the need to accumulate more clinical experience in this type of treatment.

 The major problem in cutaneous melanoma is the highly metastatic potential of the tumour and the fact that after surgery cytostatics have low efficacy. In the animal model results herein presented, using whole body mild irradiation, the antitumoral effect of the cytostastics is favoured, lowering its unwanted side effects, such as hindering the immune-based antitumoral action. Moreover, it is known that the metastatic process has a stage where tumour cells are circulating in blood/lymph, so by this therapy these cells can be better triggered with a whole body irradiation than an off-target localized one. 

## 5. Conclusions

We aimed in this study to use microwave therapy to increase the efficacy of DTIC cytostatic and monitor soluble cyto/chemokine production as possible markers for disease evolution in cutaneous melanoma animal model. Whole body irradiation with microwave was performed in an original equipment. The mice group that were subjected to combined therapy had the best clinical evolution and these findings encourage us to state that the used microwave therapy can increase the therapeutical effect of dacarbazine. We have purposely used low dacarbazine dose in order to demonstrate that in conjunction with mild total body microwave irradiation, it could contribute with positive effects to the cancer therapies. The mild total body MW irradiation and drug exposure could be a novel cutaneous melanoma therapy if MW and drug application sequences and doses are optimized and carefully controlled.

## Figures and Tables

**Table 1:** The effect of average mass (AM) of different MW exposed groups on MW absorbed power (*P*_*A*_) and SAR.

Group	AM (SD)			*P*_*A*_ (SD)			SAR (SD)		
	g			W			W/g		
	F	M	F + M	F	M	F + M	F	M	F + M
G1	21.7075 (1.0248)	24.81 (2.69293)	23.25875 (2.5116)	1.842 (0.131)	2.485 (0.515)	2.164 (0.494)	0.085 (0.002)	0.099 (0.012)	0.092 (0.011)
G2	21.5875 (1.6229)	24.775 (2.6753)	23.18125 (2.6643)	1.835 (0.200)	2.473 (0.538)	2.160 (0.519)	0.088 (0.006)	0.104 (0.017)	0.096 (0.015)
G3	22.1125 (1.1019)	23.873 (1.3832)	22.9925 (1.4918)	1.901 (0.160)	2.212 (0.592)	2.056 (0.458)	0.086 (0.003)	0.094 (0.011)	0.090 (0.009)
G4	21.6125 (1.4389)	25.15 (1.3143)	23.38125 (2.281)	1.840 (0.205)	2.627 (0.669)	2.234 (0.631)	0.085 (0.003)	0.101 (0.016)	0.094 (0.015)

Mean value	21.755 g	24.6519 g	23.2034 g	1.855 W	2.449 W	2154 W	0.086 W/g	0.100 W/g	0.093 W/g
SD of mean value	0.2439 g	0.5464 g	0.1630 g	0.0311 W	0.1729 W	0.0733 W	0.0014 W/g	0.0042 W/g	0.0026 W/g

Legend: AM: average mass over all treatment days (g); *P*_*A*_: microwave absorbed power (W); SAR = *P*_*A*_/AM; F: female; M: male; SD: standard deviation; G1: tumor-free irradiated group; G2: tumor-free irradiated + cytostatic group; G3: tumor bearing irradiated group; G4: tumor bearing irradiated + cytostatic group.
